# Teaching Anxiety, Stress and Resilience During the COVID-19 Pandemic: Evaluating the Vulnerability of Academic Professionals in Mexico Through the Adapted COVID-19 Stress Scales

**DOI:** 10.3389/fpubh.2021.669057

**Published:** 2021-05-10

**Authors:** Juan Luis Delgado-Gallegos, Gerardo R. Padilla-Rivas, Erika Zuñiga-Violante, Gener Avilés-Rodriguez, Daniel Arellanos-Soto, Hector Franco Villareal, María de los Ángeles Cosío-León, Gerardo Salvador Romo-Cardenas, Jose Francisco Islas

**Affiliations:** ^1^Departamento de Bioquímica y Medicina Molecular, Facultad de Medicina, Universidad Autónoma de Nuevo León, Monterrey, Mexico; ^2^Universidad de Montemorelos, Matamoros, Mexico; ^3^Facultad de Ingeniería, Arquitectura y Diseño, Universidad Autónoma de Baja California, Ensenada, Mexico; ^4^Althian Clinical Research, Monterrey, Mexico; ^5^Universidad Politécnica de Pachuca, Hidalgo, Mexico

**Keywords:** adapted COVID-stress scales, stress in academic professionals, resilience to COVID stress in academia, ACSS in academic professionals, anxiety during COVID

## Abstract

To mitigate the COVID-19 infection, many world governments endorsed the cessation of non-essential activities, such as the school attendance, forcing a shift of the teaching model to the virtual classroom. From this shift, several changes in the teaching paradigm derived, in addition to the COVID-19 pandemic, which could have an impact in academic professional's mental health. In the present work we show the application of a modified version of the adapted COVID-19 stress scales (ACSS) which also included teaching anxiety and preparedness, and resilience for academic professionals in Mexico. These scales were applied during the unprecedented transformation of the education system undergone in the COVID-19 quarantine. Most of the studied variables: gender, age, academic degree, household occupants, having a disease, teaching level, teaching mode, work hours, resilience, teaching anxiety and preparedness, and fear of being an asymptomatic patient (FOBAP), showed significant statistical correlation between each other (*p* < 0.050) and to the 6 areas of the ACSS (danger, contamination, social economical, xenophobia, traumatic stress, and compulsive checking). Our results further showed that the perceived stress and anxiety fell into the category of Absent to Mild, with only the danger section of the ACSS falling into the Moderate category. Finally, the resilience generated throughout the quarantine was very high, which seems to be a predictor of adaptation the academic professional has undergone to cope with stress.

## Introduction

As the COVID-19 pandemic continues to grow worldwide, the social contingency that has derived from the pandemic, has put in perspective many aspects of the daily life straining resources of different countries to the utmost capacity in terms of economic, technical, and human resources, leading to an unprecedented world crisis. One strategy to mitigate the spreading of the COVID-19, was the cessation of non-essential activities, such as school attendance. Although considered a non-essential activity, education remains vital to the advancement of society (1, 2). Therefore, there is the need to develop new strategies to mobilize the academic professional from the classroom setting to a virtual classroom, using diverse electronic platforms e.g., Zoom^®^, Microsoft TEAMS^®^, Google Classroom^®^, Moodle^®^, and others (3). Additional challenges have also arisen, in order to keep professors connected to their pupils, reduce their confusion and stress, and maintain a focus on learning (4). In developing democracies such as Mexico, internet access has added difficulties, even-though the country has made great strides in connectivity, there continues to be a struggle with affordable country wide access, making it difficult for full integration of the students to the virtual classroom (5, 6).

In Mexico, over 30 million students and two million professionals, use a variety of learning facilities each day (7). With the emergence of the COVID-19 pandemic, several academic institutions, in conjunction with the Mexican Ministry of Health issued a series of guidelines to prevent and reduce the risk of infection by COVID-19. Based on these guidelines there was the explicit directive, as of March 20th, 2020, to suspend all in-person school activities. Currently, this mandate continues being enforced (7, 8). The mandate's purpose serves to maintain afloat the educational system and help to develop it technologically, thus the need to be up-to-date in diverse technological tools and platforms, hence integrating a combination of traditional teaching and digital classroom (9, 10). However, this directly puts high levels of pressure and stress on academic professionals, particularly for those who rarely use these settings. Thus, challenging their ability to use technology and innovation to make students immerse themselves in the learning experience. From the academic professional's perspective a recent study by Martinez-Garces et al. revealed that amongst the fundamental problems academic professionals faced during the contingency with technological advancement, were ones of logistical and social-affective nature (3, 10). Researchers further mentioned that while computer information literacy is a well-developed competency, digital content creation continues to be weak (11). Therefore, it is imperative that academic professionals constantly update their computational skills such as content creation, use of digital communication tools, particularly software currently trending, in order to engage the student in the learning experience.

There are those who, because of their work, require going at least partially to a physical location, which implies the continuous use of personal protective equipment (PPE), and the combination of preparing material at a distance and in-person. As a result, this leads to physical exhaustion, emotional fatigue and the fear of infection by contact with others, hence resulting in even more anxiety and stress (12–14). This overload of physical, mental, and emotional stress can be so significant that it can trigger the development of Mild to Severe psychiatric disorders such as depression, anxiety, and even burnout syndrome. Alternatively, some studies have shown that stress can be beneficial as it helps preserve homeostasis, self-motivation, and survival. Nonetheless, stress can produce alterations in memory, cognition, learning, immune response, sleep, and both cardiovascular and endocrine system health (15–17).

An important target of treatment in depression and anxiety is resilience, a measure of coping and thriving in the face of adversity (18). We can also define resilience as a dynamic adaptative process that helps maintain a healthy psychological state. We can even say that individuals with high resilience have become mentally stronger because of adverse conditions (19).

Promoting mental health and well-being is one of the primary objectives of the WHO, particularly when it targets community development and policy making (20). In order to assess these strong and positive mental behaviors, a common strategy is the use of the resilience scale (RS-14) developed by Wagnild and Young (21, 22). This method evaluates two factors: Factor I evaluates personal competence with different factors such as self-esteem, independence, decision, wit, perseverance. Factor II evaluates life and self-acceptance through the capacity of adaptability, balance, flexibility, and stable life perspective. Typically, regional adaptations of the RS-14 are used for these assessments (22, 23). By using an adapted version of this scale, researchers in the past had measured resilience in the indigenous communities in Mexico under stressful conditions, namely after the two 2017 earthquakes. These researchers found that social support was key to develop resilience, and that among other factors both age and gender assisted. As the communities continued onward after the earthquake resilience over all became more apparent as individuals bounded even more (24). In our previous studies, we worked on understanding how stress affects the medical professionals; one of the most affected groups during the pandemic, as they are at the frontline dealing directly with the disease (14, 25). Now, we focus on evaluating the vulnerability and adaptability of the academic professionals in Mexico, as they have to face new challenges to their entire academic setting. Even though hope to end the pandemic seem near (26, 27), the potential new normal will surely have a high technological component. In this work we used the COVID-Stress Scales originally developed by Taylor el al., now adapted and applied to academic professionals (14, 28). In addition, we have now further explored resilience to the pandemic by adjusting to a section a shortened version of the RS-14 (23, 29) based on the Spanish version (24, 30) to the Academic Professional in México.

## Materials and Methods

This study explores a further application of the adapted COVID stress scales (ACSS) modified for the academic professionals in Mexico. We based this work on the ACSS by Delgado-Gallegos et al., used to evaluate stress in the daily life of medical professionals (14). Our questionnaire analyzes the six psychometric areas of the ACSS, additionally we studied resilience, teaching anxiety and preparedness, and the fear of being an asymptomatic patient (FOBAP). We added sociodemographic questions about gender, age, level of academic studies, geographic region. The full questionnaire is shown in Table 1, the original Spanish version provided as a Supplementary Material.

**Table 1:** Adapted COVID STRESS SCALES for Academic professionals in Mexico.

**Initial questions**
1 Do you want to participate in the questionnaire? ^*^ 2 What is your gender? 3 What is your age? 4 What is your academic degree? 5 State where I currently live 6 How many people live in your household, including you? 7 Do you suffer from any risky disease? 8 Academic level in which you teach 9 In what modality do you teach? 10 In case of answering in person or mixed, in the previous questionHow many hours do you spend in the physical work area per week? 11 How many hours do you work per day?
**Teaching preparedness and anxiety**
12 Do you feel concerned about the use and handling of technological tools? 13 How effective has the training you received during the health contingency period been? 14 Do you feel you have the equipment to teach virtually? 15 Are you worried about returning to the classroom in person in the coming months?
**Section 1 (Danger)**
16 I'm worried about getting the virus 17 I am concerned that basic hygiene (for example, hand washing) is not enough to keep me safe from the virus 18 I am concerned that our healthcare system cannot keep me safe from the virus 19 I'm worried that I won't be able to keep my family safe from the virus 20 I am concerned that our healthcare system may not protect my loved ones 21 I am concerned that social distancing is not enough to keep me safe from the virus
**Section 2 (Fear of Contamination)**
22 I am concerned that people around me will infect me with the virus 23 I am concerned that if I touched something in a public space (for example, handrail, handle door), you can get the virus 24 I am concerned that if someone were to cough or sneeze near me, they could catch the virus. 25 I'm worried that I might get the virus from handling money or using a card machinedebit/credit 26 I am concerned about making cash transactions 27 I am concerned that my parcel/mail has been contaminated during transit and handling. 28 I am concerned about living with people recovered from COVID-19.
**Section 3 (Social Economical)**
29 I'm worried that grocery stores will run out of food 30 I'm worried grocery stores will run out of cold or flu remedies 31 I'm concerned that pharmacies will run out of prescription drugs 32 I'm worried that grocery stores will run out of water 33 I'm worried that grocery stores will run out of cleaning products or disinfectants. 34 I'm worried about grocery stores closing 35 I'm worried about losing my job. 36 The quarantine has affected the quality of my work.
**Section 4 (Xenophobia)**
37 I'm concerned that people out of state are spreading the virus. 38 I'm concerned that people I know who live outside of my state may have the virus. 39 I'm concerned about coming into contact with people out of state because they may have the virus. 40 I'm concerned that foreign people are spreading the virus because they are not as clean as we are
41 If I went to a restaurant specializing in foreign foods, I would be worried about contracting the virus 42 If I were in an elevator with a group of foreigners, I would be concerned that they are infected with the virus.
**Section 5 (Traumatic stress)**
43 I had trouble sleeping because I was worried about the virus 44 I had bad dreams about the virus 45 I thought about the virus when I didn't want to 46 Haunting mental images about the virus appeared in my mind against my will 47 I had trouble concentrating because I kept thinking about the virus 48 Reminders of the virus caused me physical reactions, such as sweating or heartbeat strong of the heart.
**Section 6 (Compulsive Checking)**
49 I review locations on social media about COVID-19 50 I review YouTube videos about COVID-19 51 Requested peace of mind from friends or family about COVID-19 52 I check my own body for signs of infection (e.g., taking my temperature) 53 I seek advice from health professionals (for example, doctors or pharmacists) about COVID-19 54 I search the Internet for treatments for COVID-19 55 I have been diagnosed with COVID-19
**Fear of Being an asymptomatic patient**
56 I am worried about being asymptomatic and infecting my loved ones. 57 I am afraid of being reinfected with COVID-19.
**Resilience**
68 In general, I take it easy. 59 I am a person with adequate self-esteem. 60 Confidence in myself helps me get out of difficult times. 61 In an emergency, I am someone people can trust. 62 When I am in a difficult situation, I can usually find a way out.
**Final questions for future follow-up**
63 Would you be interested in taking part in a questionnaire to monitor your mental health in the future? 64 We appreciate your interest and we ask that you please leave us an email address

* *Consent to participate*.

The questionnaire was written using MS Forms^®^ (Microsoft Corporation, Redwood, WA, United States), and applied remotely through a web link. We distributed the questionnaire by electronic means to academic professionals nationwide through a partnership with the educational system in the private sector. This was done during a 1-month period in December 2020. Professionals surveyed ranged from elementary to postgraduate education teaching levels. All academic professional participating acknowledged being >18 years and gave consent by electronic means for their inclusion in the study.

We developed our questionnaire using a Likert scale system with an increasing point based system (31). Next, we tallied the results of the questionnaire and proceeded with statistical analysis correlations using IBM SPSS Statistics for Windows, version 23.0 (IBM Corp., Armonk, NY, USA) with Pearson's chi-squared ratio of 0.05.

Results from the questionnaire were then classified as described in our previous work (14, 32). Several modifications were done as Teaching Anxiety and Preparedness (four questions), and Resilience (five questions) sections were added, additionally questions were added to Contamination (seven questions), Social Economical (eight total questions), and FOBAP (two total questions). Results were classified as follows: Section with two questions Absent = 0-2, Mild = 3-4, Moderate = 5-6, Severe = 7-8. Section with four questions Absent = 0-4, Mild = 5-8, Moderate = 9-12, Severe = 13-16. Sections with six questions (original scale) Absent = 0-6, Mild = 7-12, Moderate = 13-18, Severe = 19-24. Sections with seven questions Absent = 0-7, Mild = 8-14, Moderate = 15-21, and Severe 22-28. Sections with eight questions Absent = 0-8, Mild = 9-16, Moderate = 17-24, Severe = 25-32. It is important to note that each scale can be classified independently, evaluating different aspects and variables of the daily life, and can also be evaluated together as a cumulative score. Because of the addition of questions, we made an adaptation to the scores of our previous work and added the sections of resilience and teaching anxiety and preparedness. We based the resilience section on the RS-14. The teaching, anxiety and preparedness section questions were generated to measure the level of preparedness the academic professionals had developed during the quarantine and to measure the levels of anxiety the adaptation to new methods of teaching have produced, and the return to a physical classroom using 4 questions and results were given the following classifications: Absent = 0-4, Mild = 5-8, Moderate = 9-12. Finally, we made an adaptation for the present study using five questions and we gave results using the following classifications: Very low (0-4), Low (5–8), Normal (9–12), High (13–16), and Very high (17–20). We evaluated the final version of the full questionnaire for internal consistency using Cronbach alpha (value > 0.9) to ensure reliability.

## Results

Two hundred and twenty-three participants were recruited via direct email in a collaboration with a private educational system. From the recruited participants, three declined to consent in taking part in the study. Therefore, our results for the study were calculated based on the remaining 220 participants. We should note that a potential limitation in the study was that participants were not required to answer all questions to advance through the questionnaire. The general sociodemographic information for all consenting participants is presented in Table 2.

**Table 2:** Social demographic profile of participants (*n* = 220).

**Gender**				
Male	28.5			
Female	71.04			
Others	0.45			
**Academic degree**				
High school	2.71			
Bachelor	70.13			
Graduate	27.14			
Household occupants	27.39^*^	1	>4	4
Work hours	36.15^*^	8	>8	>8
**Teaching mode**				
Presential	2.73			
Online	84.47			
Mix	7.3			
Does not apply	5.47			
**Teaching level**				
Elementary	34.86			
Junior high	24.34			
High school	22.36			
Bachelor	16.44			
Graduate	1.97			
**Diseases**				
Diabetes	7			
Cardiac	7			
Pulmonary	3			
Autoimmune	2			
Obesity	12			
Cancer	1			
HIV	<1			
Others	8			
None	60			

* *Percentage of the mode*.

**Figure 1:**
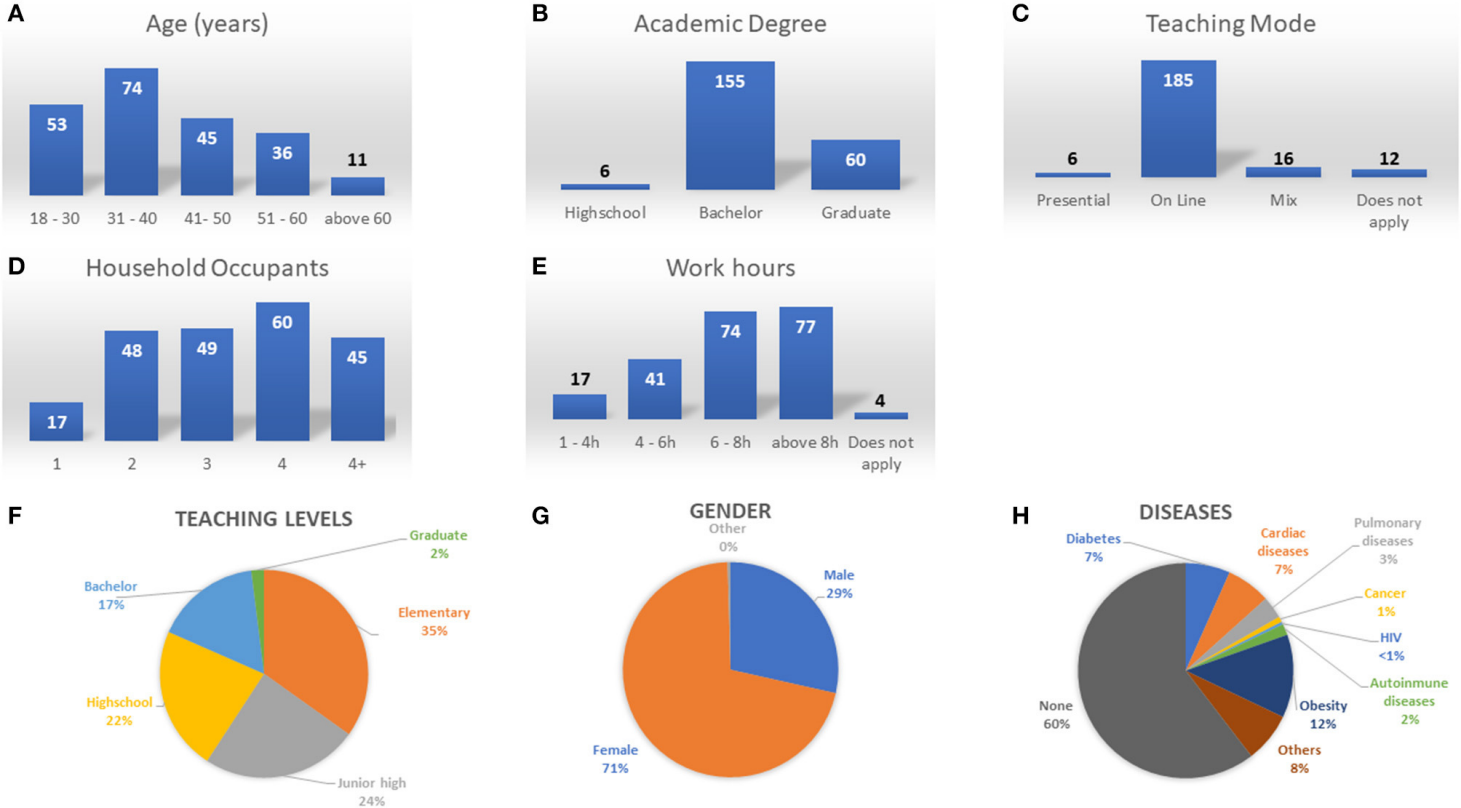
Frequency count bar graphs for **(A)** Age, **(B)** Academic degree, **(C)** Teaching mode, **(D)** Household Occupants, **(E)** Work hours. Percentage distribution for **(F)** Teaching level, **(G)** Gender, **(H)** Diseases.

Noticeably, 74 (33.78%) participants were in the age range of 31-40 years; the most frequent range, additionally 6 (2.71%) participants had a high school degree, 155 (70.13%) participants had a college degree, and 60 (27.14%) participants had a graduate degree. Participants living occupancy was for four occupants 27.39% (*n* = 60) was the most frequent answer, additionally 36.15% (*n* = 77) of participants answered working >8 h per day and 84.47% taught online. Only 2.73% reported in-person teaching and 7.3% reported mix mode teaching. Our results also showed a gender gap as there was a 3:1 ratio of Females to Males with most participants teaching at basic levels such as Elementary (34.86%), Junior high (24.34%), and High school (22.36%), while at professional 16.44% and graduate 1.97%. Several studies have shown that comorbidities can exacerbate the symptoms presented by COVID-19 (33, 34). Our frequency counts for comorbidities demonstrated that over 60% of responders did not present any comorbidities, 12% mentioned having at least obesity and 7% diabetes. In addition, we observed cardiac diseases in 7% of the participants and Pulmonary diseases in 3%. Figure 1 shows frequency counts for social demographic variables and percentage distributions.

**Figure 2:**
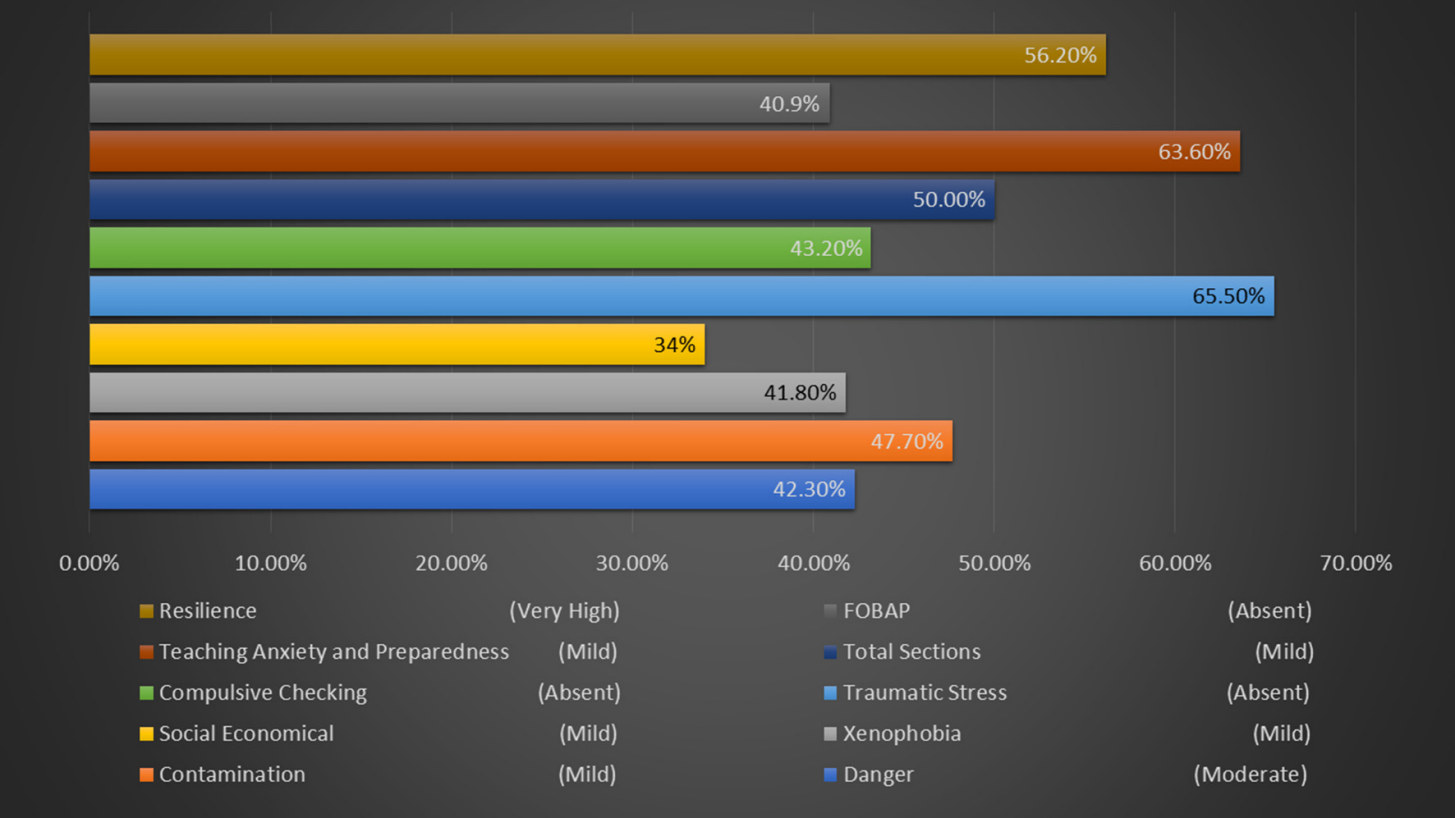
Most representative COVID-related stress frequency (shown as percentage) for the ACSS, Teaching Anxiety and Preparedness, and Resilience for Academic Professionals.

Next, we evaluated stress frequency as interpreted by the ACSS for the six sections to understand into which classification most academic professionals fall into. Our results for the most frequent classification in ACSS areas showed: Danger (Moderate 42.3%, *n* = 93), Contamination (Mild 47.7%, *n* = 105), Xenophobia (Mild 41.8%, *n* = 92), Social Economical (Mild 34%, *n* = 74), Traumatic Stress (Absent 65.5 %, *n* = 144), Compulsive Checking (Absent 43.2%, *n* = 95), and for the Total sections (Mild 50%, *n* = 110). We further tested for Teacher Anxiety and Preparedness (Mild 63.6%, *n* = 140) and FOBAP (Absent 40.9%, *n* = 90). Additionally, frequency counts for resilience classifications showed that most academic professionals had very high resilience (56.2%, *n* = 118). Table 3 shows the full COVID related stress frequency and classification, and Figure 2 graph percentages for most representative frequencies.

**Table 3:** COVID-related stress frequency for the ACSS, teaching anxiety and preparedness, and resilience for academic professionals.

	**Danger**		**Contamination**		**Xenophobia**		**Social economical**	
	***N***	**%**	***N***	**%**	***N***	**%**	***N***	**%**
Absent	21	9.5	46	20.9	47	21.4	38	17.5
Mild	70	31.8	405	47.7	92	41.8	74	34
Moderate	90	42.3	59	26.8	66	30	72	33.1
Severe	36	16.4	10	4.5	15	6.8	33	15.4
Total		100		100		100		100
	**Traumatic stress**		**Compulsive checking**		**Total sections**			
	***N***	**%**	***N***	**%**	***N***	**%**		
Absent	144	65.5	95	43.2	37	16.9		
Mild	49	22.3	82	37.3	110	50		
Moderate	18	8.2	32	14.5	35	29.5		
Severe	9	4.1	11	5	8	3.6		
Total		100		100		100		
	**Teaching anxiety and preparedness**		**FOBAP**					
	***N***	**%**	***N***	**%**				
Absent	14	6.4	90	40.9				
Mild	140	63.6	76	34.5				
Moderate	65	29.5	40	18.2				
Severe	1	0.5	14	6.4				
Total		100		100				
	**Resilience**							
	***N***	**%**						
Very low	10	4.7						
Low	4	2						
Neutral	21	9.9						
High	67	31.9						
Very high	118	56.2						
Total		100						

Since our primary goal was to study how COVID-19 has affected academic professionals, we analyzed the statistical correlation between all survey variables. All correlations are shown on Table 4. In summary, female participants showed correlations (*p* < 0.001) to all variables (Resilience, Teaching Anxiety and Preparedness, FOBAP, Danger, Contamination, Social Economical, Xenophobia, Traumatic stress, Compulsive checking, Total Sections), while males showed no correlation to Social Economical (*p* < 0.076) (Supplementary Table 1). From the age ranges, ages 18 to 30 years showed correlation to all variables except Social Economical (*p* < 0.298), 31 to 40 years and 41 to 50 years showed statistical correlation to all variables, 51 to 60 years showed correlation to all variables except for Social Economical (*p* < 0.774) and Contamination (*p* < 0.076). Finally, participants above 60 years only showed correlations to Resilience (*p* < 0.029), Teaching Anxiety and Preparedness (*p* < 0.004), and FOBAP (*p* < 0.035) (Supplementary Table 2). For Academic degree, participants with only a high school degree did not show correlations to any of the studied variables, for those with a professional degree and above statistical correlations were seen in all variables (Supplementary Table 3). For Household occupants, 1-person occupancy showed statistical relevance with FOBAP (*p* < 0.010), Contamination (*p* < 0.039), and Traumatic stress (*p* < 0.007), for those living with an additional companion statistical correlation were seen in all variables except for Social Economical (*p* < 0.771). Interestingly, individuals living in households of 3 or 4 additional companions results were statistically correlated to all variables, yet individual living with 4 or more companions the variables of FOBAP (*p* < 0.066) and Social Economical (*p* < 0.057) were not statistically relevant (Supplementary Table 4). Expectantly, participants with a comorbidity showed statistical relevance to all variables (Supplementary Table 5). Academic professionals teaching at an Elementary, Junior High School, High School, and Bachelor levels showed statistical relevance to all variables, meanwhile those teaching at a Graduate level did not show any correlations (Supplementary Table 6). For those teaching in-person, no correlation was shown in any variable. For professionals teaching online statistical relevance was seen in all variables. Strikingly, professionals teaching in a mix form show correlation only to Teaching Anxiety and Preparedness (*p* < 0.039), Traumatic stress (*p* < 0.001), Compulsive checking, and Total sections (*p* < 0.002) (Supplementary Table 7). For those professionals working for up to 4 h, results showed statistical relevance only to Resilience (*p* < 0.047), Teaching Anxiety and Preparedness (*p* < 0.023), and both Danger and Compulsive checking (*p* < 0.028). For those professionals working from 4 to 6h, results were relevant to all variables except for Social Economical (*p* < 0.191). For those professionals working 6 or more hours all variables presented statistical correlations (Supplementary Table 8).

**Table 4:** Statistical correlations of all variables: resilience, teaching anxiety and preparedness, FOBAP, Sociodemographic profiles, ACSS and Total Sections.

	**Resilience**	**Teaching anxiety and preparedness**	**FOBAP**	**Danger**	**Contamination**	**Social economical**	**Xenophobia**	**Traumatic stress**	**Compulsive checking**	**Total sections**
Resilience		*p* < 0.631	*p* < 0.185	***p<*** **0.001**	***p<*** **0.004**	***p<*** **0.021**	***p<*** **0.002**	***p<*** **0.007**	***p<*** **0.020**	***p<*** **0.001**
Teaching anxiety and preparedness	*p* < 0.631		*p* < 0.356	***p<*** **0.001**	*p* < 0.100	***p<*** **0.007**	***p<*** **0.003**	*p* < 0.491	*p* < 0.175	***p<*** **0.001**
FOBAP	*p* < 0.185	*p* < 0.356		***p<*** **0.001**	***p<*** **0.001**	***p<*** **0.001**	***p<*** **0.001**	***p<*** **0.001**	***p<*** **0.001**	***p<*** **0.006**
Gender										
Females	***p<*** **0.001**	***p<*** **0.001**	***p<*** **0.001**	***p<*** **0.001**	***p<*** **0.001**	***p<*** **0.001**	***p<*** **0.001**	***p<*** **0.001**	***p<*** **0.001**	***p<*** **0.001**
Males	***p<*** **0.001**	***p<*** **0.001**	***p<*** **0.001**	***p<*** **0.001**	***p<*** **0.001**	*p* < 0.076	***p<*** **0.002**	***p<*** **0.001**	***p<*** **0.001**	***p<*** **0.001**
Age										
18 to 30 y	***p<*** **0.001**	***p<*** **0.001**	***p<*** **0.001**	***p<*** **0.010**	***p<*** **0.001**	*p* < 0.298	***p<*** **0.001**	***p<*** **0.001**	***p<*** **0.001**	***p<*** **0.001**
31 to 40 y	***p<*** **0.001**	***p<*** **0.001**	***p<*** **0.001**	***p<*** **0.003**	***p<*** **0.001**	***p<*** **0.001**	***p<*** **0.001**	***p<*** **0.001**	***p<*** **0.001**	***p<*** **0.001**
41 to 50 y	***p<*** **0.001**	***p<*** **0.001**	***p<*** **0.004**	***p<*** **0.001**	***p<*** **0.001**	***p<*** **0.036**	***p<*** **0.001**	***p<*** **0.001**	***p<*** **0.031**	***p<*** **0.001**
51 to 60 y	***p<*** **0.002**	***p<*** **0.001**	***p<*** **0.001**	***p<*** **0.042**	*p* < 0.076	*p* < 0.774	***p<*** **0.042**	***p<*** **0.001**	***p<*** **0.003**	***p<*** **0.001**
Above 60 y	***p<*** **0.029**	***p<*** **0.004**	***p<*** **0.035**	*p* < 0.234	*p* < 0.484	*p* < 0.631	*p* < 0.200	*p* < 0.178	*p* < 0.147	*p* < 0.529
Academic degree										
High school	*p* < 0.655	*p* < 0.819	*p* < 0.819	*p* < 0.449	*p* < 0.449	*p* < 0.449	*p* < 0.655	*p* < 0.655	*p* < 0.449	*p* < 0.819
Bachelor	***p<*** **0.001**	***p<*** **0.001**	***p<*** **0.001**	***p<*** **0.001**	***p<*** **0.001**	***p<*** **0.001**	***p<*** **0.001**	***p<*** **0.001**	***p<*** **0.001**	***p<*** **0.001**
Graduate	***p<*** **0.001**	***p<*** **0.001**	***p<*** **0.001**	***p<*** **0.001**	***p<*** **0.001**	***p<*** **0.022**	***p<*** **0.001**	***p<*** **0.001**	***p<*** **0.001**	***p<*** **0.001**
State of Residence	*p* < 0.995	*p* < 0.676	***p<*** **0.048**	*p* < 0.084	*p* < 0.102	*p* < 0.145	*p* < 0.233	***p<*** **0.013**	*p* < 0.205	***p<*** **0.013**
Household occupants	*p* < 0.555	***p<*** **0.015**	*p* < 0.488	*p* < 0.060	*p* < 0.459	*p* < 0.725	*p* < 0.867	*p* < 0.443	*p* < 0.696	*p* < 0.326
Diseases										
w/ comorbidity	***p<*** **0.001**	***p<*** **0.001**	***p<*** **0.001**	***p<*** **0.001**	***p<*** **0.001**	***p<*** **0.003**	***p<*** **0.001**	***p<*** **0.001**	***p<*** **0.002**	***p<*** **0.001**
Teaching levels										
Elementary	***p<*** **0.001**	***p<*** **0.001**	***p<*** **0.001**	***p<*** **0.001**	***p<*** **0.001**	***p<*** **0.019**	***p<*** **0.001**	***p<*** **0.001**	***p<*** **0.001**	***p<*** **0.001**
Junior High school	***p<*** **0.001**	***p<*** **0.001**	***p<*** **0.001**	***p<*** **0.001**	***p<*** **0.001**	***p<*** **0.002**	***p<*** **0.001**	***p<*** **0.001**	***p<*** **0.001**	***p<*** **0.001**
High school	***p<*** **0.001**	***p<*** **0.001**	***p<*** **0.001**	***p<*** **0.001**	***p<*** **0.001**	***p<*** **0.050**	***p<*** **0.001**	***p<*** **0.001**	***p<*** **0.001**	***p<*** **0.001**
Bachelor	***p<*** **0.001**	***p<*** **0.001**	***p<*** **0.001**	***p<*** **0.029**	***p<*** **0.001**	*p* < 0.084	***p<*** **0.001**	***p<*** **0.001**	***p<*** **0.001**	***p<*** **0.001**
Graduate	*p* < 1.000	*p* < 0.414	*p* < 1.000	*p* < 0.607	*p* < 0.102	*p* < 0.607	*p* < 0.607	*p* < 0.223	*p* < 0.223	*p* < 0.223
Teaching mode										
In-person	*p* < 0.100	*p* < 0.607	*p* < 0.414	*p* < 0.607	*p* < 0.607	*p* < 0.607	*p* < 0.223	*p* < 0.607	*p* < 0.607	*p* < 0.607
Online	***p<*** **0.001**	***p<*** **0.001**	***p<*** **0.001**	***p<*** **0.001**	***p<*** **0.001**	***p<*** **0.001**	***p<*** **0.001**	***p<*** **0.001**	***p<*** **0.001**	***p<*** **0.001**
Mix	*p* < 0.305	***p<*** **0.039**	*p* < 0.090	*p* < 0.058	*p* < 0.058	*p* < 0.090	*p* < 0.321	***p<*** **0.001**	***p<*** **0.029**	***p<*** **0.002**
Work hours	*p* < 0.485	*p* < 0.439	*p* < 0.800	*p* < 0.152	*p* < 0.788	*p* < 0.065	*p* < 0.333	*p* < 0.199	*p* < 0.254	*p* < 0.077

*Values with statistical significance (p < 0.05)*.

Finally, we analyzed Resilience, Teaching Anxiety and Preparedness, and FOBAP between themselves and the ACSS. As expected, Resilience showed statistical correlation to the ACSS, yet not to either Teaching Anxiety and Preparedness (*p* < 0.631) or FOBAP (*p* < 0.185) (Supplementary Table 9). Teaching Anxiety and Preparedness showed statistical relevance to Contamination (*p* < 0.001), Social Economical (*p* < 0.007), Xenophobia (*p* < 0.003), and Total Sections (*p* < 0.001) (Supplementary Table 10). Lastly, FOBAP, showed correlations to the whole ACSS and Total Sections (Supplementary Table 11).

## Discussion

The nature of academics is to prepare young minds for the challenges of today and for the world of tomorrow. Interestingly, our results show that Teaching Anxiety and Preparedness, Social Economical, Contamination, Xenophobia and Total sections resulted in Mild. Statistically, Teaching Anxiety and Preparedness result correlated with having an academic degree, hence showing that having a higher degree (higher preparedness) could be an indicator of less stress. Even though, online teaching has continued now for two periods (semesters) stress continues, particularly as professionals continue to struggle to have students fully engaged in their activities, most likely due to internet accessibility issues (1, 35, 36).

We noticed that the Social Economical stress indicator of the ACSS did not correlate with males, nor with the youngest or oldest professionals. In addition, these indicators were highly relevant to having a comorbidity (potential to exacerbate by COVID-19), working with young (non-professional level) students, and at least working 6 h per day. As mentioned earlier, the herculean task is not only to teach young minds, but to maintain them engaged. Unlike professional level students (or Graduates), were recording lectures is an advantage, younger minds are more prone to household distractions.

We found that only in a handful of cases such as having a high school diploma only, or living alone, resilience did not show any statistical correlation, meaning that adaptability to stress has been achieved by much of the academic professionals (18). In this particular study, as we mentioned we modeled a section for resilience based on the RS-14 (24, 29, 30). In a case study in Mexico, researchers had previously evaluated the levels of resilience in indigenous populations after the 2017 earthquakes finding that resilience develops overtime and is further enhance by social integration (24), after nearly a year since the beginning of the pandemic we can draw a parallel between the indigenous populations of the aforementioned and the academic professionals, as in both cases we see high levels of resilience. This suggest that although social distancing has been put in place, digital communications have permitted academic professionals adapt their teaching model (3, 11).

From the results of the recollected sociodemographic profile data, as seen on Table 2 and the statistical relevance as shown in Table 4, People >50 years old seem to have lower anxiety and stress related to COVID-19, with high levels of resilience. Could resilience develop over a lifetime? Or is the age-old phrase “wisdom comes with age” hold true, particularly for academic professionals. Recently, Pearman et al. denoted that proactive coping was a protective measure against COVID-19 related stress. As older patients are more prone to have complications if infected. Hence, they have a more hands-on attitude toward complying with restrictive measures and even developing positive habits based on earlier experience (37).

As stated earlier, having a college degree seems to be a determining factor correlating with all variables. Much like experience, education seems to play a role in mental well-being. Although, at a personal level social anxiety can induce stress, education ensures preparedness; a minimum requirement in today's ever more challenging economy. Particularly, we stand in an environment where technology is key. For academic professionals we have seen that distance educating is the norm, and that challenges arise with young students for engagement, hence it is no surprise that with better preparation, professional's strive becoming less stressed (1, 10).

Another interesting variable survey was the amount of household occupants. Understandably, participants with two or more household occupants seem to develop more stress than 1 occupant, which can be explained because of the preoccupation of getting sick through a companion or even getting infected from one another. Studies has shown that other factors such as food waste in time of stress is exacerbated by the number of occupants, inevitably putting more strain on the household as a unit (38). Expectantly, participants with at least 1 comorbidity, showed a statistical correlation to all the studied variables, which can be understood given that they can be more susceptible to developing a Severe presentation of COVID-19 (33, 34).

In our study, academic professionals taught mostly (>80%) at elementary school, junior high school, and high school, additionally another >16% taught a bachelor level with just <2% teaching at a graduate level. Interestingly, all but the latter had a strong correlation to all studied variables. Given that the graduate students do not need as much tutoring as a kid or teenager, graduate professors do not develop as much stress. The most affected participants were the ones doing online teaching, due to the transformation of in-person teaching to a virtual classroom and the growing demands of the students and schoolwork (1, 3, 39). Understandably, professionals working more hours (>6 h) tend to develop more stress than the people working 0 to 4 h a day. Resilience, teaching anxiety and preparedness, and FOBAP, have significant correlation to the ACSS, which is understandable, given that it closely relates to the anxiety and stress mechanisms (40). Our results further showed that total stress in many categories resulted in Absent to Mild, while only the danger category presented Moderate, and none presented Severe. In this study we further analyzed resilience, which demonstrated to be very high for academic professionals. Compared to our previous study, where we analyzed healthcare professionals attending COVID-19, our results showed that most cases they presented Mild to Moderate stress with traumatic stress, compulsive checking and xenophobia being the most affected areas, along with FOBAP (14).

Education is highly-related to a country's advancement, therefore reducing the levels of stress and procuring better resources for academic professional should be a priority. Therefore, Key strategies to prevent the development of stress and anxiety should include job security, accessibility to internet and maintenance of hardware, continuous education in electronic platforms and pedagogic resources, vaccination program priority, access to medical services and psychological support.

## Conclusion

In the present work we have shown the application of the ACSS for academic professionals during the unprecedented transformation of the education system undergone in the COVID-19 quarantine. This has made a significant impact on the work routine of the vast majority of academic professionals. Most of the variables studied: gender, age, academic degree, household occupants, having a disease, teaching level, teaching mode, work hours, resilience, teaching anxiety and preparedness, and FOBAP show significant statistical correlation, to each other and the six areas of the ACSS (danger, contamination, social economical, xenophobia, traumatic stress and compulsive checking) which can be translated into a development of Mild to Moderate perceived stress and anxiety in the daily life, caused by COVID-19. Although, the high relevance of COVID-19 induced stress and anxiety, the resilience generated during the process of the transformation of the educational system, can be a predictor of the adaptation the academic professional has undergone during the quarantine to cope with stress.

Although the “n” seems limited, it shows the behavior of this specific population. Due to the COVID-19 quarantine and social distancing restrictions, the application of the questionnaire was done throughout a digital platform (MS FORMS). Even-though this could be considered a limitation as it is not applied and supervised in-person, this remote evaluation provides a safe alternative, thus relying on the participant's willingness to respond. Nonetheless, a study with more academic professionals is needed to address the rising mental health pandemic due to COVID-19.

## Data Availability Statement

The original contributions presented in the study are included in the article/Supplementary Material, further inquiries can be directed to the corresponding author's.

## Ethics Statement

The studies involving human participants were reviewed and approved by Comite de Etica, Hospital La Misión, Monterrey Nuevo León. The patients/participants provided their written informed consent to participate in this study.

## Author Contributions

JD-G and JFI: research and writing. GP-R and EZ-V: statistical analysis. GA-R and DA-S: conceptualization. HF-V and MC-L: resources. GR-C and JFI: supervision. All authors contributed to the article and approved the submitted version.

## Conflict of Interest

The authors declare that the research was conducted in the absence of any commercial or financial relationships that could be construed as a potential conflict of interest.

## Supplementary Material

Supplementary Table 1 Statistical Correlations for Gender (by Females and Males) vs. Adapted COVID STRESS SCALES, Teaching Anxiety and Preparedness, Resilience, and FOBAP.

Supplementary Table 2 Statistical Correlations for Age (by groups) vs. Adapted COVID STRESS SCALES, Resilience, Teaching Anxiety and Preparedness, and FOBAP.

Supplementary Table 3 Statistical Correlations for Academic degree held (High School, Bachelor, Graduate) vs. Adapted COVID STRESS SCALES, Teaching Anxiety and Preparedness, Resilience, and FOBAP.

Supplementary Table 4 Statistical Correlations for the number of Household occupants vs. Adapted COVID STRESS SCALES, Teaching Anxiety and Preparedness, Resilience, and FOBAP.

Supplementary Table 5 Statistical Correlations of having a Disease (comorbidity) vs. Adapted COVID STRESS SCALES, Teaching Anxiety and Preparedness, Resilience, and FOBAP.

Supplementary Table 6 Statistical Correlations of academic levels (Teaching levels) taught vs. Adapted COVID STRESS SCALES, Teaching Anxiety and Preparedness, Resilience, and FOBAP.

Supplementary Table 7 Statistical Correlations for Teaching mode (in-person, online, and mix) vs. Adapted COVID STRESS SCALES, Teaching Anxiety and Preparedness, Resilience, and FOBAP.

Supplementary Table 8 Statistical Correlations for Work hours (by range) vs. Adapted COVID STRESS SCALES, Teaching Anxiety and Preparedness, Resilience, and FOBAP.

Supplementary Table 9 Statistical Correlations for Resilience vs. Adapted COVID STRESS SCALES, Teaching Anxiety and Preparedness, and FOBAP.

Supplementary Table 10 Statistical Correlations for Teaching Anxiety and Preparedness vs. Adapted COVID STRESS SCALES, and FOBAP.

Supplementary Table 11 Statistical Correlations for FOBAP vs. Adapted COVID STRESS SCALES.
