# Supplementary material for: Teaching Anxiety, Stress and Resilience During the COVID-19 Pandemic: Evaluating the Vulnerability of Academic Professionals in Mexico Through the Adapted COVID-19 Stress Scales
Source: Front Public Health. 2021 May 10;9:669057. doi: 10.3389/fpubh.2021.669057 (PMC8141807; doi:10.3389/fpubh.2021.669057)
Supplement: Supplementary Table 1 — Statistical Correlations for Gender (by Females and Males) vs. Adapted COVID STRESS SCALES, Teaching Anxiety and Preparedness, Resilience, and FOBAP. [file Data_Sheet_1.ZIP › Suppplement Table Spanish.docx]

Inicial Questions

1 Desea usted participar en el cuestionario?*

2 Cual es su genero?

3 Cual es su edad?

4 Cual es su grado académico?

5 Estado en donde vide actualmente

6 Cuantas personas viven en su casa, incluyendo usted?

7 Padece usted alguna enfermedad de riesgo?

8 Nivel académico en el que imparte clase

9 En que modalidad imparte clase?

10 En caso de contestar presencial o mixto, en la pregunta anterior

¿Cuántas horas pasa usted en el area física de trabajo a la semana?

11 Cuantas horas trabaja al dia?

Teaching Preparedness and Anxiety

12 Se siente preocupado por el uso y manejo de las herramientas tecnológico?

13 Qué tan efectiva ha sido la capacitación que recibido durante el periodo de la contingencia sanitaria?

14 Siente que tiene el equipo necesario para dar clases de forma virtual?

a

15 Le preocupa regresar al aula de forma presencial en los próximos meses?

Section 1 (Danger)

16 Estoy preocupado por contraer el virus

17 Me preocupa que la higiene básica (por ejemplo, el lavado de manos) no sea suficiente para mantenerme a salvo del virus

18 Me preocupa que nuestro sistema de salud no pueda mantenerme a salvo del virus

19 Me preocupa no poder mantener a mi familia a salvo del virus

20 Me preocupa que nuestro sistema de salud no pueda proteger a mis seres queridos

21 Me preocupa que el distanciamiento social no sea suficiente para mantenerme a salvo del virus

Section 2 (Fear of Contamination)

22 Me preocupa que las personas a mi alrededor me infecten con el virus

23 Me preocupa que si tocara algo en un espacio público (por ejemplo, pasamanos, manija

de la puerta), pueda contraer el virus

24 Me preocupa que si alguien tosiera o estornudara cerca de mí, podría contraer el virus.

25 Me preocupa que pueda contraer el virus al manejar dinero o usar una máquina de tarjeta de débito/crédito

26 Estoy preocupado por hacer transacciones en efectivo

27 Me preocupa que mi paquetería / correo haya sido contaminado durante su tránsito y manejo.

28 Me preocupa convivir con personas recuperadas de COVID-19.

Section 3 (Socialeconomical)

29 Me preocupa que las tiendas de comestibles se queden sin comida

30 Me preocupa que las tiendas de comestibles se queden sin remedios para el resfriado o la gripe

31 Me preocupa que las farmacias se queden sin medicamentos recetados

32 Me preocupa que las tiendas de comestibles se queden sin agua

33 Me preocupa que las tiendas de comestibles se queden sin productos de limpieza o desinfectantes.

34 Me preocupa que las tiendas de comestibles cierren

35 Me preocupa perder mi trabajo.

36 La cuarentena ha afectado la calidad de mi trabajo.

Section 4 (Xenophobia)

37 Me preocupa que personas fuera del estado estén propagando el virus.

38 Me preocupa que las personas que conozco, que viven fuera de mi estado, puedan tener el virus.

39 Me preocupa entrar en contacto con personas fuera del estado porque pueden tener el virus.

40 Me preocupa que personas extranjeras estén propagando el virus porque no están tan limpios como nosotros

41 Si fuera a un restaurante especializado en alimentos extranjeros, me preocuparía contraer el virus

42 Si estuviera en un elevador con un grupo de extranjeros, me preocuparía que estén infectados con el virus.

Section 5 (Traumatic stress)

43 Tuve problemas para dormir porque me preocupaba el virus

44 Tuve malos sueños sobre el virus

45 Pensé en el virus cuando no quise

46 Aparecieron en mi mente, contra mi voluntad, imágenes mentales inquietantes sobre el virus

47 Tuve problemas para concentrarme porque seguía pensando en el virus

48 Los recordatorios del virus me provocaron reacciones físicas, como sudoración o latidos

fuertes del corazón.

Section 6 (Compulsive Checking)

49 Reviso ubicaciones en redes sociales sobre COVID-19

50 Reviso videos de YouTube sobre COVID-19

51 Solicitó tranquilidad a amigos o familiares sobre COVID-19

52 Reviso mi propio cuerpo en busca de signos de infección (p. Ej., Tomando mi temperatura)

53 Pido consejo a los profesionales de la salud (por ejemplo, médicos o farmacéuticos)

sobre COVID-19

54 Busco en Internet tratamientos para COVID-19

55 He sido diagnosticado con COVID-19

Fear of Beaing an asymptomatic patient

56 Estoy preocupado de ser asintomático y contagiar a mis seres queridos.

57 Tengo miedo de reinfectarme con COVID-19.

Resilience

68 En general, me tomo las cosas con calma.

59 Soy una persona con adecuada autoestima.

60 La seguridad en mí mismo, me ayuda a salir de momentos difíciles.

61 En una emergencia soy alguien en quien la gente puede confiar.

62 Cuando estoy en una situación difícil por lo general puedo encontrar una salida.

Final questions for future follow-up

63 ¿Le interesaría en un futuro participar en un cuestionario para seguimiento de su salud mental?

64 Le agradecemos su interés y le pedimos, por favor nos deje una dirección de correo electrónico

* Consentimiento a participar
